# *Retracted*: CD248 as a novel therapeutic target in pulmonary arterial hypertension

**DOI:** 10.1002/ctm2.488

**Published:** 2021-07-16

The following article from *Clinical and Translational Medicine*, “CD248 as a novel therapeutic target in pulmonary arterial hypertension” by Tao Xu, Lei Shao, Aimei Wang, Rui Liang, Yuhan Lin, GuanWang, Yan Zhao, Jing Hu, and Shuangyue Liu,^1^ published online on September 17, 2020 in Wiley Online Library (wileyonlinelibrary.com), has been retracted by agreement between the authors, the journal Editor‐in‐Chief, Xiangdong Wang and John Wiley & Sons, Ltd. The retraction has been agreed due to duplicate use of figures and data in the article.
